# Behavioral Account of Attended Stream Enhances Neural Tracking

**DOI:** 10.3389/fnins.2021.674112

**Published:** 2021-12-13

**Authors:** Moïra-Phoebé Huet, Christophe Micheyl, Etienne Parizet, Etienne Gaudrain

**Affiliations:** ^1^Laboratoire Vibrations Acoustique, Institut National des Sciences Appliquées de Lyon, Université de Lyon, Villeurbanne, France; ^2^CNRS UMR 5292, INSERM U1028, Auditory Cognition and Psychoacoustics Team, Lyon Neuroscience Research Center, Lyon, France; ^3^Starkey France S.a.r.l., Creteil, France; ^4^Department of Otorhinolaryngology, University Medical Center Groningen, University of Groningen, Groningen, Netherlands

**Keywords:** neural tracking, attentional switches, temporal response function (TRF), speech-on-speech, vocal cues

## Abstract

During the past decade, several studies have identified electroencephalographic (EEG) correlates of selective auditory attention to speech. In these studies, typically, listeners are instructed to focus on one of two concurrent speech streams (the “target”), while ignoring the other (the “masker”). EEG signals are recorded while participants are performing this task, and subsequently analyzed to recover the attended stream. An assumption often made in these studies is that the participant’s attention can remain focused on the target throughout the test. To check this assumption, and assess when a participant’s attention in a concurrent speech listening task was directed toward the target, the masker, or neither, we designed a behavioral listen-then-recall task (the Long-SWoRD test). After listening to two simultaneous short stories, participants had to identify keywords from the target story, randomly interspersed among words from the masker story and words from neither story, on a computer screen. To modulate task difficulty, and hence, the likelihood of attentional switches, masker stories were originally uttered by the same talker as the target stories. The masker voice parameters were then manipulated to parametrically control the similarity of the two streams, from clearly dissimilar to almost identical. While participants listened to the stories, EEG signals were measured and subsequently, analyzed using a temporal response function (TRF) model to reconstruct the speech stimuli. Responses in the behavioral recall task were used to infer, retrospectively, when attention was directed toward the target, the masker, or neither. During the model-training phase, the results of these behavioral-data-driven inferences were used as inputs to the model in addition to the EEG signals, to determine if this additional information would improve stimulus reconstruction accuracy, relative to performance of models trained under the assumption that the listener’s attention was unwaveringly focused on the target. Results from 21 participants show that information regarding the actual – as opposed to, assumed – attentional focus can be used advantageously during model training, to enhance subsequent (test phase) accuracy of auditory stimulus-reconstruction based on EEG signals. This is the case, especially, in challenging listening situations, where the participants’ attention is less likely to remain focused entirely on the target talker. In situations where the two competing voices are clearly distinct and easily separated perceptually, the assumption that listeners are able to stay focused on the target is reasonable. The behavioral recall protocol introduced here provides experimenters with a means to behaviorally track fluctuations in auditory selective attention, including, in combined behavioral/neurophysiological studies.

## Introduction

Popularized by Cherry (1953) as the “cocktail-party problem” over 60 years ago, the question of how human listeners selectively attend a speaker amid one or several other concurrent voices, has attracted considerable interest to this day. While recent developments in machine-learning algorithms now allow machines to compete with – and in some situations, overtake – humans in this ability, a complete account of the psychological and neurophysiological processes at play remains elusive. Nonetheless, during the past decade, significant progress toward elucidating brain-activity correlates of the perceptual experience of listening selectively to one of two concurrent voices has been achieved. In particular, researchers have been able to identify features of electrically or magnetically recorded cortical signals which, after mathematical transformation, exhibit greater correlation with features of the target voice, than with features of the competing, non-target voice (e.g., Ding and Simon, 2012; Mesgarani and Chang, 2012; O’Sullivan et al., 2015).

One limitation of most earlier studies using the concurrent voice paradigm to study neural correlates of selective auditory attention, however, stems from their use of an experimental design in which participants were asked to attend to the target voice, and ignore the concurrent voice, over prolonged periods – from a few minutes to several tens of minutes. The premise that human listeners are able to unwaveringly maintain their auditory attention focused on a single sound source, be it a human voice, for such long time periods is at odds with introspective experience while participating in such – somewhat artificial – listening experiments involving concurrent voices. Our own experience, and informal reports from participants, strongly suggest that despite one’s best efforts to stay focused on the target voice, the competing voice occasionally grabs one’s attention. Unless such occasional attentional shifts can be controlled for, they can adversely impact data-analysis methods used to assess neural representations of the attended voice. Specifically, temporal response functions (TRFs) are obtained by relating temporal sequences of stimuli to the continuous brain activity recorded in response to them, by means of machine learning methods. Typically, for two competing speech streams, the temporal envelope of either of the streams is used to either predict the brain activity (*forward* TRF), or to be predicted by the brain activity (*backward* TRF). One of the differences between these two approaches is that forward TRFs treat each neural response channel independently while backward TRFs exploit the whole neural data in a multivariate context (Crosse et al., 2016). In addition, backward approaches can predict or “decode” which of the speakers the listener is attending. For this reason, backward TRFs are often called “*decoders*” whereas the accuracy to classify which speaker is attended is commonly referred as “*decoding accuracy*.” Misestimating which of the competing streams is actually attended can impact the accuracy of these decoding algorithms in two ways. First, they can interfere with the training of the algorithm, if the brain responses used for training span epochs during which the participant was actually attending to the non-target voice. Second, they can interfere with the measured decoding accuracy of the algorithm at test-time, if the brain responses on which the algorithm is tested are assumed to contain only target-attend, or only non-target-attend, epochs.

To mitigate this issue, some investigators have made attempts to assess the occurrence of attentional shifts during the experiment. For instance, O’Sullivan et al. (2015) asked their listeners multiple-choice questions following every 1-min stimulus, to check that the listener had been paying attention to the target story. One limitation of this approach, however, is that listeners may have been able to answer the questions correctly, even if they did not always pay close attention to the target stream. Crosse et al. (2015) asked participants to press a button whenever they were listening to the target voice. However, it is possible that most listeners are unable to, simultaneously, perform the demanding listening task, and to accurately report their auditory-attention status accurately. Moreover, asking listeners to press buttons according to their attention while they are listening introduces a secondary task, which may perturb performance in the primary, selective-attention task. The problem of attention shifts has been acknowledged, and attempts to develop attention-decoding algorithms that can cope with such shifts have been developed (Akram et al., 2016, 2017; Miran et al., 2018; Jaeger et al., 2020). However, except when a distraction was purposely inserted (Holtze et al., 2021), most studies in the literature seem to have remained limited by the almost complete lack of detailed data regarding the timing of attentional shifts in selective-listening experiments with concurrent voices.

The issue of attentional switching across two concurrent auditory streams can hardly be approached without considering how easy, or hard, it is for listeners to perceptually separate these two streams. Previous studies have shown that two of the most important cues used by human listeners to separate concurrent voices are spatial separation and differences in the fundamental-frequency (F0) or timbre of these voices (Bronkhorst, 2015; Middlebrooks, 2017). Recently, two studies have investigated attentional switching with spatial cues. Bednar and Lalor (2020) showed that it was possible to reconstruct, with TRFs, the trajectory of attended and unattended moving sound sources. In the second paper, carried by Teoh and Lalor (2019), participants had to focus on a target voice while both talkers (target and masker) were instantaneously alternated between the left and right ears. The authors showed that it was possible to significantly improve the auditory attention decoding accuracy with the inclusion of spatial information.

In the present work, we investigate the assumption that listeners are able to maintain their attention focused on the target speech stream and assess when a participant’s attention was directed toward the target, the masker, or neither with a test that was designed to provide experimenters with a means of inferring fluctuations in auditory selective attention: the Long-SWoRD test (Huet et al., 2021). Here, participants’ answers are used to infer, retrospectively, when they were listening to the target, or to the masker. The difficulty of the task, and hence the likelihood of an increase in attentional switch to occur during the course of the stories, was modulated with vocal cues. The attention course was modeled by combining the participants’ responses with three different parameters. These parameters, described in section “‘Inferred Stimuli,” depict the speed and duration of attentional switches as well as the actual sound source that receives the focus of attention. This better representation of actual attended speech is expected to yield a better stimulus-reconstruction evaluation since it takes into account attentional dynamics.

## Materials and Methods

### Participants

Twenty-one participants, aged between 19 and 25 (μ = 21 years, σ = 1.76), participated in the experiment. All of them were native French speakers and had audiometric thresholds ≤ 30 dB HL at audiometric test frequencies between 125 Hz and 8 kHz. Participants gave informed consent before taking part in the study and were paid an hourly wage for participation.

### Procedure

The Long-SWoRD test (Huet et al., 2021) was used to obtain estimates of the attended stream at different time points of the stimulus. Two competing stories were presented diotically at the same time to both ears. Participants were instructed to focus on one of the two concurrent speech streams (the “target”), while ignoring the other (the “masker”). At the end of the trial, nine keywords, arranged in a three-by-three matrix, were presented on a screen facing the participant. The three rows corresponded, from top to bottom, to the beginning, middle and end portions of the story. Each row included, in a random order, one keyword from the target sentence, one keyword from the “masker” sentence, and an “extraneous” keyword which was contained neither in the target nor in the masker sentence. Participants were instructed to select the three keywords in the target story with the constraint that they could select only one keyword in each row.

The difficulty of the task, and therefore the probability of attentional switches occurring, was modulated by manipulating the perceptual distance between the two competing stories in terms of voice (see section “Voice Manipulation”). The experiment was arranged into 12 blocks, randomly distributed between three levels of difficulty. Within each block, there were 12 trials and the same distance between the target and the masker voices was kept. The characteristics of these voices are described in the next section.

Data collection lasted 60–100 min, and the entire procedure was completed in a single session. Participants were instructed to avoid eye movements to reduce potential noise in the electroencephalographic (EEG) recording. Stimuli were presented with OpenSesame (Mathôt et al., 2012). Participants listened to stimuli diotically over Sennheiser HD250 Linear II headphones in a sound-attenuated booth. The presentation level was calibrated to 65 dB SPL using an AEC101 artificial ear and sound level meter LD824 (Larson Davis, Depew, NY, United States).

### Stimuli

#### Material Content

This material was previously developed and used in two behavioral studies (Huet et al., 2018, 2021). Short, interesting and engaging stories, extracted from the French audiobook “*Le Charme discret de l’intestin*” (The Inside Story of Our Body’s Most Underrated Organ) (Enders et al., 2015), provided the stimulus set for the target and masker streams. Each story was composed of 1–5 sentences. Each trial, composed of a target story and a masker story of similar length, lasted between 11 and 18 s.

The three target keywords that participants would later have to identify were selected at three different times within the story: one keyword near the beginning of the story, one keyword toward the middle of the story, and one keyword toward the end. The same selection procedure was applied for the masker keywords, whereas the extraneous keywords originate from other trials. Further details and considerations about the choice of keywords and statistical analyses of the linguistic features of the stimuli can be found in Huet (2020) and Huet et al. (2021).

#### Voice Manipulation

Manipulating the parametric distance in semitones (st) between the target and the masker voices and thus, varying the difficulty level of the task is an approach used in previous experiments (e.g., Darwin et al., 2003; Vestergaard et al., 2009; Ives et al., 2010; Başkent and Gaudrain, 2016). The audio stimuli were originally recorded by an adult female speaker. This original voice, analyzed and resynthesized without modification (i.e., with a voice distance of 0 st) with the STRAIGHT toolbox (Kawahara et al., 1999) implemented in MATLAB, was chosen as the target voice. For the creation of the three masker voices, the voice pitch (F0) and vocal-tract length (VTL) were then manipulated during the analysis-resynthesis. The total distance between the target and the masker voices is then calculated, in semitones, as $\sqrt{△\,F\,0^{2}+△\,V\,T\,L^{2}}$. Total distance values of 1.14 st, 3.42 st, and 5.13 st were chosen to constitute three levels of masking, difficult, intermediate, and easy, respectively. In a previous experiment (Huet et al., 2021), we were able to estimate that a difference of 5.13 st is a good control condition since the participants made almost no error as a result of the large voice difference. In addition, participants did not make more masker errors than extraneous errors, suggesting no target-masker confusions and no or almost no attentional switches. Parameter values for the three masker voices are provided in Table 1.

**TABLE 1:** Distance between the target and the masker voices, in semitones.

**Condition**	**ΔF0**	**ΔVTL**	$\sqrt{△\,{\text{F0}}^{2}+△\,{\text{VTL}}^{2}}$
Difficult	−1.6	0.4	1.14
Intermediate	−3.2	1.21	3.42
Easy	−4.8	1.82	5.13

### “Inferred” Stimuli

**FIGURE 1:**
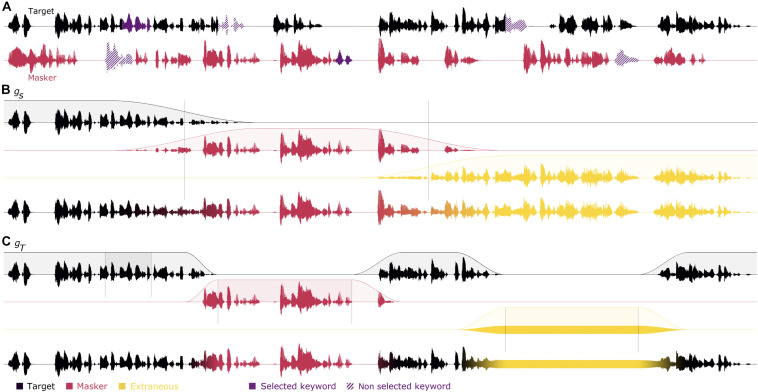
Creation steps of inferred stimuli. Panel **(A)** represents an example of a trial where the target is in black and the masker is in red. In this example, the participant has answered the first target keyword (highlighted in purple), the second masker keyword and the third extraneous keyword. The unselected keywords are shown with a hatched pattern. **(B)** The three segments are built according to the participant’s answer with an attention switch of 3 s and the extraneous segments filled with the mixture of the target and masker (i.e., method *h*_+_, in yellow). The attention scopes are interpolated with the segment method *g*_*S*_. The three segments are then added together. **(C)** The three attention scopes are built according to the participant’s answer with an attention switch of 1 s and the extraneous sections filled with noise (*h*_*N*_). When no behavioral information is known, the attention scope is filled with the target (i.e., *g*_*T*_). The three parts are then added together.

“Inferred stimuli” are reconstructed stimuli derived from the original stimuli and from the behavioral responses aiming at estimating the actual attended stream, switching between target and masker streams. Participants’ responses provide information at three key moments in the story (beginning, middle, and ending keywords). It is important to note that there are not just three keywords that provide information, but six: three target and three masker keywords. For each response, the participants cannot choose both target and masker, but are faced with a choice. Thus, if a participant selects the masker keyword in a line of the matrix, it is possible to hypothesize that the subject was not listening to the associated target keyword. The information is therefore not limited in time to the chosen keyword, but also extended to the associated non-selected keyword in the other stream, and which may not be occurring exactly at the same time. Thus, there are three key moments in the stories, bound by the time limits of the target and the masker keywords, which provide information. For convenience, these key moments will be named “windows.” For instance, in Figure 1A, the first target and masker keywords overlap while conversely, the second target and masker keywords are separated by 1 s. Therefore, these key moments, or windows, can have varying durations. In addition, the windows duration started at the beginning of the keyword that appears first, and ended at the end of the keyword that ended last. Based on the participants’ answers, the inferred stimuli were modeled following various strategies differing in how three aspects of the task were handled: the duration and speed of attentional switches (described respectively in sections “Extrapolation of Attentional Scope” and “Attentional Switch Speed”) as well as the sound source to which attention is focused (described in section “Extraneous Keywords Fillers”).

Mathematically, the inferred stimulus can be expressed as follows:


$$\hat{x}\,\left(t\right)=\left\{ \begin{array}{c} f\,\left(x_{T},x_{M},R_{i}\right)\,\left(t\right)\,\text{if}\,t\in \mathrm{keyword}\,\mathrm{window}\,i\\ g\,\left(x_{T},x_{M}\right)\,\left(t\right)\,\text{otherwise} \end{array}\right.$$

Where *x*_*T*_ and *x*_*M*_ are the target and masker stimuli, respectively; *R*_*i*_ designates the response to keyword *i*; and *f* is the following function:


$$f\,\left(x_{T},x_{M},R_{i}\right)=\left\{ \begin{array}{c} x_{T}\,\text{if}\,R_{i}\,\mathrm{is}\,\mathrm{target}\,\mathrm{keyword}\\ x_{M}\,\text{if}\,R_{i}\,\mathrm{is}\,\mathrm{masker}\,\mathrm{keyword}\\ h\,\left(x_{T},x_{M}\right)\,\text{otherwise} \end{array}\right.$$

The functions *g* and *h* are defined below.

#### Extrapolation of Attentional Scope

Outside of the windows defined by the keyword positions, since there is no behavioral data collected, the attentional locus is not explicitly known and needs to be inferred. This situation occurs for instance at the very beginning of the stories (before the first keyword), at the end of the stories (after the last keyword), and between consecutive windows. Two different approaches were used to estimate attention outside of the windows, thereafter referred to as *attentional scopes*.

In the first approach “*segments*,” illustrated in Figure 1B, the stimulus duration was divided into three segments based on the temporal positions of the keywords. The cut-out points between segments were placed in time halfway between two consecutive windows, or at the beginning and end of the stimulus. In this case, we are making the assumption that the attention information provided in the windows remains over a wider duration than the window itself, dividing the unknown segments equally across windows. This corresponds to a function *g* as follows:


$$g_{s}\,\left(x_{T},x_{M}\right)\,\left(t\right)=f\,\left(x_{T},x_{M},R_{i}\right)\,\left(t\right),$$

where *R*_*i*_ is the closest known response.

In the second approach “*target windows*,” illustrated in Figure 1C, it is assumed that the information contained in the window should be limited to the window itself only, which is the opposite of the first approach, which extended the information to an entire segment. Between windows, it then can be assumed that the participant was always listening to the same stream (either the target or the masker). Figure 1C illustrates a situation where the participant listens to the target outside of the windows. The cases where the listener always listens to the target in-between keywords correspond to the following variant of the *g* function:


$$g_{T}\,\left(x_{T},x_{M},R_{i}\right)=x_{T}$$

This parameter will be referred to as “scope” with the function *g*_*S*_ “segments” and the function *g*_*T*_ “target windows.”

#### Extraneous Keywords Fillers

In addition to attending to the target, or to the competing (non-target) voice, participants in concurrent-voice experiments may also, at times, not be attending to either. Thus, in addition to inferring when attention was directed to the target or to the masker, it is also important to try to infer when it is not. To this aim, participants’ selection of displayed keywords that belonged to neither of the two stories played during the trial, i.e., the extraneous keywords, is instrumental. The selection of an extraneous keyword over a keyword from either story may be an indication that, when the keywords that the participant failed to select were presented, the participant was not attending to either of the two stories being played. In such a case, neither the target nor the masker is more appropriate than the other stream to represent the attended stimulus. One way of representing this situation thus consists in using the mixture of the two streams as attended stimulus (illustrated in Figure 1B). This, corresponds to a function *h* as follows:


$$h_{+}{}_{\left(x_{T},x_{M}\right)=x_{T}+x_{M}}$$

This might account for situations where the listeners were actually dividing their attention between the two streams, which led them to fail to recall the corresponding keyword at the end of the trial. However, it is also possible that the listeners, in these situations, were actually not attending *any* of the presented streams. In these situations, the mixture does not seem the most appropriate acoustic correlate of what the participant is focusing on, and instead, a random noise signal (illustrated in Figure 1C, noted *h*_*N*_) has been used to fill in the extraneous keywords. Additionally, a target speech story from another trial of the Long-SWoRD corpus (noted *h*_*S*_), randomly selected, was also used as a control speech signal. The root-mean-square level of the extraneous fillers have been adapted to match the root-mean-square level of the target. Finally, for completeness, we also considered the case where extraneous keyword responses would be treated as the target (*h*_*T*_) or masker (*h*_*M*_) streams:


$$\begin{array}{c} h_{T}\,\left(x_{T},x_{M}\right)=x_{T}\\ h_{M}\,\left(x_{T},x_{M}\right)=x_{M} \end{array}$$

This parameter will be referred to as “filler.”

#### Attentional Switch Speed

The third parameter is the speed with which participants can switch from one voice to another. The duration of this attentional switch is modeled as the slope of the edges of the time windows. Three values were used, 1, 2, and 3 s, implemented as raised-cosine ramps. Those values were chosen as they could capture attentional switches: slower than speech modulations, but shorter than sentences to limit overlap across segments. This parameter will be referred to as “speed.”

### Data Acquisition and Signal Processing

**FIGURE 2:**
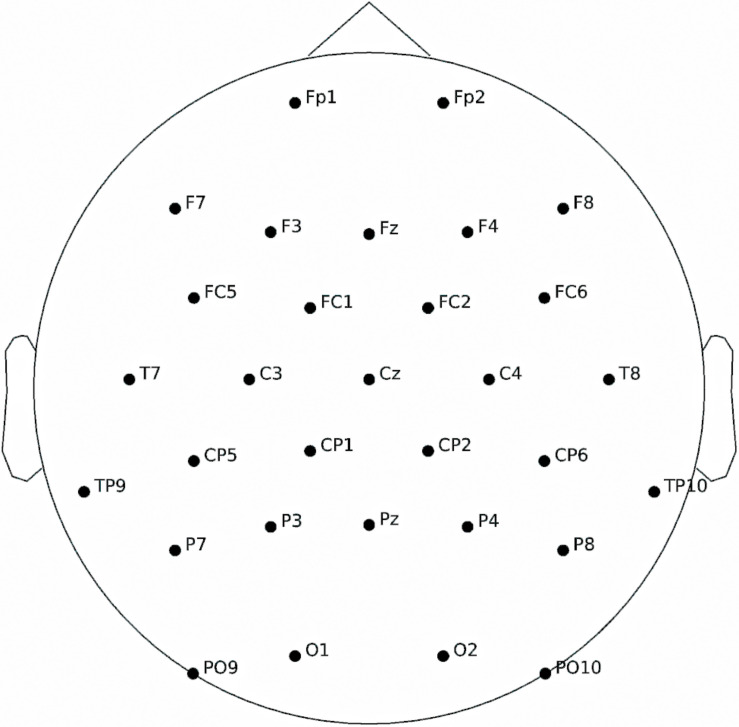
Electroencephalographic channel positions.

Electroencephalographic data were recorded using an ActiCap (Brain Products, Munich, Germany) with a setup of 31 channels at a sampling rate of 1000 Hz (see Figure 2 for more information). A trigger was sent at each start of a new trial on a parallel port with a precision of 1 ms. EEG data were then band-pass filtered between 2 and 8 Hz. Finally, to decrease processing time, EEG data were downsampled to 64 Hz.

The stimulus speech envelope was extracted with a gammatone filterbank (Søndergaard et al., 2012; Søndergaard and Majdak, 2013) followed by a power law according to Biesmans et al. (2017). The gammatone filterbank was composed of 28 bands centered on frequencies from 50 to 5000 Hz, equally spaced on the ERB_*N*_ scale (Glasberg and Moore, 1990). The envelopes of each frequency band were extracted by taking the absolute value and then raising it to the power of 0.6. A single envelope for the stimulus was then computed by averaging the 28 envelopes. The speech envelope was then downsampled to 64 Hz and low-pass filtered below 8 Hz, following the method described by O’Sullivan et al. (2015).

### Backward Modeling and Stimulus-Reconstruction

Regularized linear regression was employed to relate the neural data to the envelopes and the decoders were calculated using the MNE-Python library (Gramfort et al., 2014), using the backward method, i.e., reconstructing the audio envelope from the EEG recording. These decoders, equivalent to backward TRFs, are composed of weights that can be estimated by a linear regression for a set of *N* electrodes at different lags *t*. In this experiment, we investigated time lags between −900 to 0 ms (meaning the audio could precede the EEG up to 900 ms) by steps of 1 sample of the EEG recording (15.6 ms). Therefore, the EEG data were cut according to the duration of the trials added with the lags *t*. As for speech envelopes, they were padded with zeros to match the number of samples of EEG data. Finally, the reconstruction of the speech envelope ${\hat{S}}_{t}$can be obtained as follows:


$${\hat{S}}_{t}=\sum_{n=1}^{N}\sum_{\tau }d_{\tau ,n}\,R_{t-\tau ,n}$$

Where **R** represents the matrix that contains the shifted neural responses of each electrode *n* at time *t* = 1…*T*. A ridge regression was used to obtain the weights of the *decoder d* as follows:


$$d={\left(R\,R^{\text{T}}+\lambda \,\text{I}\right)}^{-1}\,\left(R\,S^{\text{T}}\right)$$

where λ is the regularization parameter, chosen to optimize the stimulus-response reconstruction, ***I*** is the identity matrix, and ***S*** is the envelope of the speech signal. The optimal ridge parameter was fit with an adaptive procedure according to Crosse et al. (2016) and set to 10^1/2^. Decoders were estimated per trial, for each subject in each condition. The stimulus-reconstruction of a single trial was predicted in a leave-one-out fashion. To be more precise, each subject had 48 trials per condition. Each trial was reconstructed with the averaged decoder trained on the 47 other trials. The stimulus-reconstruction was evaluated with the Pearson’s correlation coefficient between the reconstructed speech envelope and the original speech envelope. This reconstruction accuracy is thereafter noted *r*. The temporal resolution of the reconstructed envelope was the same as that of the original envelope (64 Hz).

### Statistical Analyses

All statistics were performed using R (R Core Team, 2017). All the linear mixed models (LMMs) were implemented with the *lme4* package (Bates et al., 2014). The models were implemented using a top-down strategy on data (Zuur et al., 2009). The final model is reported with the *lme4* syntax such as Equation 1:


(1) $$\text{Score}∼{\text{factor}}_{\text{A}}\times {\text{factor}}_{\text{B}}+\left({\text{factor}}_{\text{A}}\times {\text{factor}}_{\text{B}}|\text{subject}\right)$$

The full-factorial model is indicated by the fixed effect term *factor*_*A*_ × *factor*_*B*_ and includes main effects and interactions for these two main conditions. The last term of the equation describes an individual random intercept and slope per subject for *factor*_*A*_ and *factor*_*B*_. *Factor*_*A*_, *factor*_*B*_, and *Score* will be specified in section “Results” for each analysis.

For an easier interpretation, the *afex* package (Singmann et al., 2019) was used to compute the statistics of main effects. To do so, the final model was compared to restricted models in which the effect estimated is fixed and equal to zero. Finally, *post hoc* analyses were computed with a false discovery rate (FDR) correction (Benjamini and Hochberg, 1995).

## Results

### Behavioral

**FIGURE 3:**
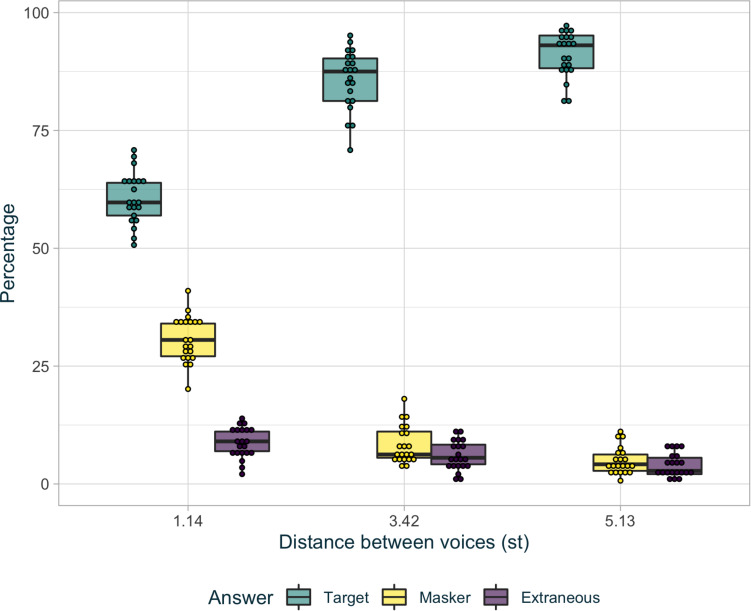
Percentage for target answers (in green), masker answers (in light yellow), and extraneous answers (in dark purple) in each level of difficulty. The points represent each identified keywords percentage for every participant in each condition. The hinges of the boxplot represent the first and the third quartile. The middle of the boxplot is the median. The whiskers extend up to 1.5 times the interquartile range.

Figure 3 shows the percentage of identified keywords (“target,” “masker,” and “extraneous”) for each level of voice difficulty (difficult: 1.14 st; intermediate: 3.42 st; and easy: 5.13 st). A generalized linear mixed model (gLMM) was fitted on the binary (correct/incorrect) scores. Such models are well suited to preserve homoscedasticity and to minimize the effects of saturation in binomial data. For each keyword within each trial, if the participant selected the target keyword, the score was positive (i.e., score correct = 1). On the other hand, if the participant selected the masker keyword or the extraneous keyword, the score was considered incorrect (i.e., score incorrect = 0).

Equation 2 shows the final model with a top-down strategy modeling:


(2) Score∼voice+(voice|subject)

Participants had better scores when the distance between the target and the masker voices was larger (2.13,*SE* = 0.12,*z* = 18.02,*p* < 0.001). *Post hoc* analysis showed that average scores in each voice condition were all different from one another. In addition, there were significantly more masker responses than extraneous responses when stimuli were only presented with the 1.71 st voice (*z* = 13.06, *p* < 0.001), but not for a voice distance of 3.42 st (*z* = 1.24, *p* = 0.22), or 5.13 st (*z* = 0.09, *p* = 0.93). These results indicate that participants were listening, at least partially, to the masker voice instead of the target voice only in the difficult condition whereas the switches between target and masker were limited in the two other conditions.

### Stimulus-Reconstruction Evaluation

#### Modeling Parameters

Because the parameter space defining the possible inferred stimuli is rather larger, before comparing it to the original approach, we selected the set of parameters that gave the best reconstruction.

**FIGURE 4:**
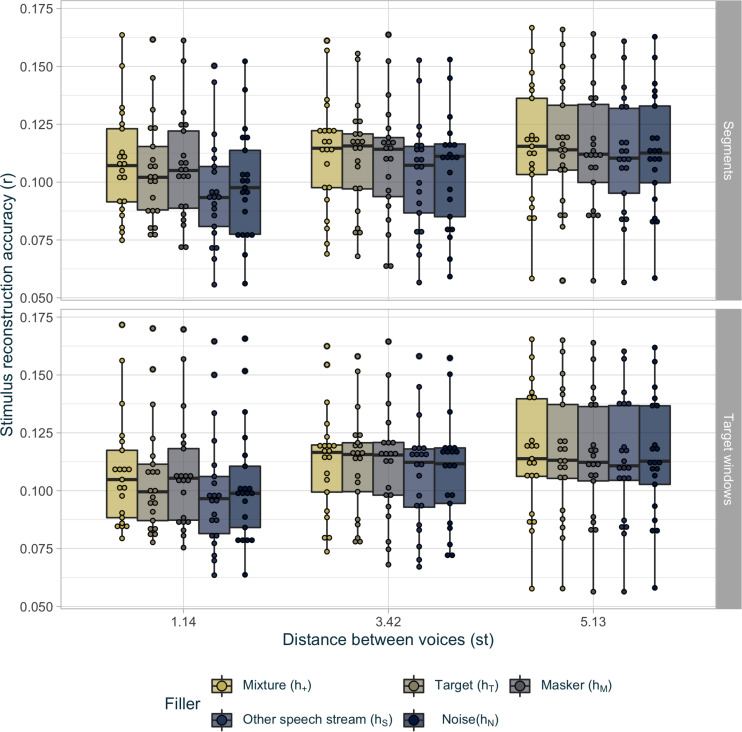
Average reconstruction accuracy *r* for inferred envelopes per condition. The points represent the scores for every participant in each voice condition for the extraneous keyword (in color) and the attentional scope (top and bottom). The hinges of the boxplot represent the first and the third quartile. The median is represented as a bar in each boxplot. The whiskers extend over 1.5 times the interquartile range.


Figure 4 shows stimulus reconstruction accuracy in each voice condition as a function of extrapolation method (scope: segments *g*_*S*_ or target windows *g*_*T*_) and treatment method for the extraneous keywords (filler : mixture *h*_+_, target *h*_*T*_, masker *h*_*M*_, and other speech stream *h*_*S*_ or noise *h*_*N*_), averaged across speed of attentional switch (speed: 1, 2, or 3 s). The influence of the three modeling parameters (scope, filler, and speed) as well as the voice distance (difficult: 1.14 st; intermediate: 3.42 st; and easy: 5.13 st) was also analyzed with a LMM fitted to the Fisher transformed Pearson’s correlation *r* values representing the reconstruction accuracy. Equation 3 indicates the final model:

(3) r∼voice⁢×⁢filler+scope+(1|subject)

Similarly to the analysis in the previous section, the distance between voices had an effect on reconstruction performance (χ^2^(2) = 282.16,*p* < 0.001) but post-doc analyses with a FDR correction did not identify any individual difference between the conditions (see Table 2). Regarding the modeling parameters, the filler for the extraneous keyword (*h*_*T*_, *h*_*M*_, *h*_+_, *h*_*N*_, or *h*_*S*_) had an effect on the reconstruction of the inferred stimuli (χ^2^(4) = 100.59,*p* < 0.001). *Post hoc* analyses, detailed in Table 2, showed that when the time segment corresponding to extraneous keyword responses was filled-up with the mixture (*h*_+_), stimulus reconstruction accuracy was the best. The target (*h*_*T*_) and masker (*h*_*M*_) streams were second-best, followed by the noise (*h*_*N*_) and, lastly, the other speech (*h*_*S*_) stream. The interaction between the latter two factors (χ^2^(8) = 25.17,*p* < 0.01) showed an effect of the filler, only when the distance between the two voices was 1.14 st (χ^2^(3) = 78.04,*p* < 0.001) and 3.42 st (χ^2^(3) = 38.38,*p* < 0.001) but not for 5.13 st (χ^2^(3) = 8.71,*p* = 0.07). Furthermore, in this easiest voice condition (5.13 st), the reconstruction accuracy performed with the target (*h*_*T*_) as the filler reached the reconstruction accuracy performed with the mixture (*h*_+_) [*t*(20) = 0.29,*p* = 0.77] while the reconstruction accuracy performed with the masker (*h*_*M*_) was equivalent to the reconstruction accuracy performed with the noise (*h*_*N*_) (*t*(20) = −0.39,*p* = 0.74). Finally, the attentional scope (*g*_*T*_ and *g*_*s*_) also had a significant effect (χ^2^(1) = 9.23,*p* < 0.01), with a better performance when the target-windows approach (*g*_*T*_) was used over the three segments (*g*_*s*_) (*t*(20) = −3.13,*p* < 0.01). It is noteworthy than the attentional switch (1, 2, or 3 s) speed had no effect on reconstruction performance [χ^2^(2) = 0.45,*p* = 0.50].

**TABLE 2:** *Post hoc* analyses for Equation 3.

**Main effect**	**Individual comparison**	**Statistics**
Difference between voices	1.14 st vs. 3.42 st	*t*(20) = −1.82,*p* = 0.13
	1.14 st vs. 5.13 st	*t*(20) = −2.52,*p* = 0.06
	3.42 st vs. 5.13 st	*t*(20) = −1.17,*p* = 0.26
Extraneous keyword filler	Mixture vs. target	*t*(20) = 5.26,*p* < 0.001
	Mixture vs. masker	*t*(20) = 5.43,*p* < 0.001
	Target vs. masker	*t*(20) = 0.65,*p* = 0.53
	Target vs. other speech stream	*t*(20) = 8.11,*p* < 0.001
	Target vs. noise	*t*(20) = 6.54,*p* < 0.001
	Other speech stream vs. noise	*t*(20) = −6.64,*p* < 0.001

In conclusion, the best stimulus-reconstruction evaluation was obtained when the behavioral response was used only at the location of the keywords and the remaining segments were filled with the target stream (*g*_*T*_), while the mixture was used in case of extraneous keyword responses (*h*_+_), regardless of the attentional switch duration. In the following section, the term “behavioral decoder” denotes a decoder obtained using these best parameters (*g*_*T*_, *h*_+_) and a 2-s attentional switch duration.

#### Target vs. Inferred Stimulus

In this section, the best behavioral decoder is compared with the original target decoder. The evaluation of these two decoders was analyzed with a LMM fitted on the Fisher-transformed *r* values representing reconstruction accuracy:


(4) r∼voice×decoder+(voice|subject)

**FIGURE 5:**
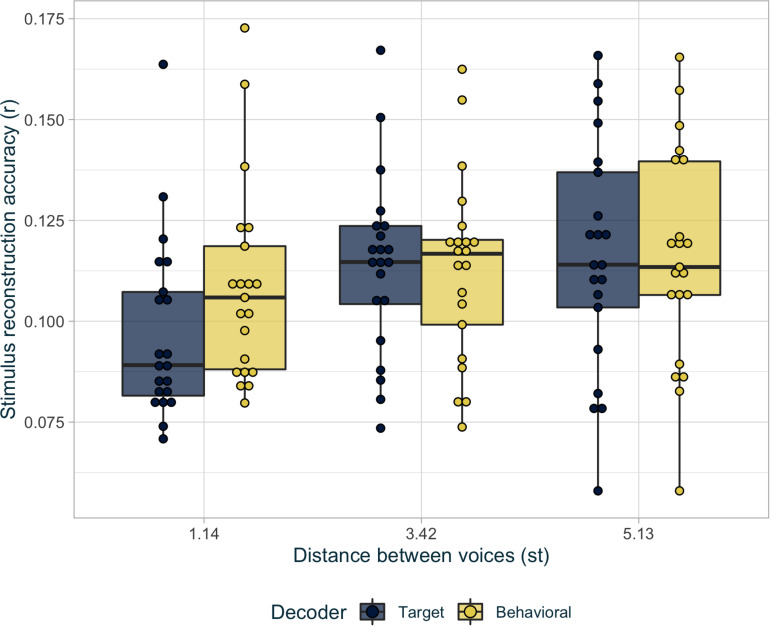
Average reconstruction accuracy *r* per condition for the decoder based on original target stimuli (in dark blue), and for the decoder based on the best inferred stimuli (in light yellow).

Based on likelihood-ratio tests, stimulus reconstruction accuracy depended significantly on the distance between the two voices (χ^2^(2) = 11.95,*p* < 0.01), on whether the target or behavioral decoder was used (χ^2^(2) = 11.1,*p* < 0.001), and the interaction between these two factors (χ^2^(2) = 28.85,*p* < 0.001). In *post hoc* comparisons, the behavioral decoder was significantly superior to the original target decoder in the most challenging voice condition, while there was no difference between decoders for the two easier voice conditions (see Figure 5 and Table 3). In addition, unlike reconstruction with the target decoder, there was no difference in performance between the voice conditions when reconstructing with inferred stimuli (see Table 3).

**TABLE 3:** *Post hoc* analyses for the Equation 4 interaction.

**Individual comparison**	**Statistics**
1.14 st: target vs. behavioral decoder	*t*(20) = −4.6,*p* < 0.001
3.42 st: target vs. behavioral decoder	*t*(20) = 0.76,*p* = 0.54
5.13 st: target vs. behavioral decoder	*t*(20) = 0.62,*p* = 0.54
Target decoder: 1.14 st vs. 3.42 st	*t*(20) = −4.55,*p* < 0.01
Target decoder: 1.14 st vs. 5.13 st	*t*(20) = −4.04,*p* < 0.01
Target decoder: 3.42 st vs. 5.13 st	*t*(20) = −0.54,*p* = 0.6
Behavioral decoder: 1.14 st vs. 3.42 st	*t*(20) = −1.4,*p* = 0.27
Behavioral decoder: 1.14 st vs. 5.13 st	*t*(20) = −1.64,*p* = 0.23
Behavioral decoder: 3.42 st vs. 5.13 st	*t*(20) = −0.64,*p* = 0.6

## Discussion and Conclusion

In this work, we assessed whether the reconstruction accuracy of attention-decoding algorithms in a selective-listening task can be enhanced by making use of information regarding the time-course of attentional shifts inferred using participants’ answers in a keyword-recall task performed immediately after a selective-listening task. The answer to this question was found to be positive. Consistent with our hypotheses, an advantage of the new decoding method, including estimates of the timing of attention shifts, was only observed in challenging listening conditions, where the participants’ attention was less likely to remain focused on the target talker throughout the entire listening-trial duration; no improvement over the simpler decoding algorithm, which did not make use of information regarding the timing of attentional shifts during the trial, was found for the easy listening conditions.

These findings are particularly relevant for future applications of the attention-decoding paradigm. While most experimental work thus far has focused on normal-hearing (NH) listeners attending to clearly separated speech streams (most often, two speakers of different genders presented dichotically), one of the ultimate goals of this line of research is to use attention decoding to enhance speech perception for hearing impaired (HI) listeners in challenging situations. However, HI listeners do not benefit as much as NH listeners from voice differences in competing speech (e.g., Festen and Plomp, 1990), and the situation seems even more severe for cochlear implant (CI) users (e.g., Pyschny et al., 2011; El Boghdady et al., 2019). In the present study, we show that, not only can stimulus reconstruction accuracy still be performed under conditions where voice cues are not salient, but it can also be improved further, to the point that decoding performance in difficult listening conditions can equal performance in easy listening conditions – provided that the decoder is trained with stimuli that account for the behavioral responses of the participant that indicate attention switches.

### Lack of Benefit in Less Challenging Conditions

The lack of stimulus-reconstruction enhancements in easy conditions can be explained by a reduced number of errors from participants. Two to three times more errors were made in the challenging condition than in the other conditions. Since inferred stimuli are based on the participants’ answers, in a trial where no error occurs in the behavioral task, the inferred stimulus is identical to the original target. It is therefore not surprising that under easy conditions, minimal differences between the inferred stimuli and the original target reconstructions were observed. However, it remains unclear whether this lack of errors truly reflects a lack of attentional switch between competing speech streams, or lack of sensitivity of the behavioral procedure used here; specifically, the procedure may have failed to capture momentary shifts in attention in-between keywords. Indeed, in connected speech, perceptual, and cognitive compensation, a process sometimes referred to as *phonemic restoration*, can help a listener infer missing segments (Bashford et al., 1992). It is possible that the participants’ attention sometimes wavered away from the target, but that they still managed to infer the correct response in the task nonetheless. However, the Long-SWoRD test was designed such as to limit the possibility of such restoration mechanisms. First, the target and masker sentences both came from the same audiobook, and had largely overlapping lexical fields. In addition, the extraneous keywords were chosen to be equally likely to occur in the context of the target and the masker – see Huet et al. (2021), for a detailed analysis of the material. Given these methodological-design precautions, it seems less likely that phonemic restoration played a major role in compensating for momentary attention switches; it seems more likely that attention switches remained very limited.

### Optimal Parameters for Inferred Stimuli

Several parameters were used to model the inferred stimuli. Extraneous keyword filling seems to be the most important factor, with an improved reconstruction in a challenging condition when the extraneous keyword is replaced by the mixture (*h*_+_), the target (*h*_*T*_) [*t*(20) = −3.84,*p* < 0.01] or the masker (*h*_*M*_) [*t*(20) = −3.88,*p* < 0.01] stream compared to noise (*h*_*N*_) [*t*(20) = −1.85,*p* = 0.08]or another story (*h*_*S*_) [*t*(20) = −0.85,*p* = 0.4]. These results suggest that when participants failed to select the target keyword, or the masker keyword (if they mistakenly switched to the masker stream), they still listened to the presented speech streams. This could be because the failure to choose the target or masker keyword was caused by a failure to recall the correct word, rather than by a failure to attend the speech streams. Alternatively, it could be that, even when the listener’s attention was directed elsewhere than to the target stream, primary automatic speech processes induced large-enough synchronous EEG activity to support reliable stimulus reconstruction accuracy. If so, using noise or an unrelated speech stimulus would necessarily lead to lower reconstruction accuracy. Further insight into this question may be gained by considering that no difference in reconstruction accuracy was noted, depending on whether extraneous keywords were replaced with targets (*h*_*T*_) or with maskers (*h*_*M*_) in challenging conditions. This result further suggests that, for these segments for which extraneous keywords were selected by participants, the participants were either dividing their attention across the two streams or listening to the mixture; this provides further justification for using the mixture as a filler (*h*_+_). In addition, a difference in reconstruction was observed in the easiest listening condition when the extraneous keywords were filled with targets (*h*_*T*_) or maskers (*h*_*M*_): the reconstruction was improved with targets (*h*_*T*_), to the point of matching a reconstruction performed with mixtures (*h*_+_) as fillers. This finding suggests that it is reasonable to consider that participants have no (or nearly none) attentional switches when the difference between the target and masker voices is large enough.

The attentional scope extrapolation method, which was used to infer where the attention was directed in-between and around keywords, also influenced reconstruction accuracy. Inferred stimuli that modeled that the participant listens to the target even outside the keyword windows achieved a better reconstruction; this again suggests that it may be reasonable to assume that listeners are able to stay focused on the target throughout the trial.

### Effect Size of the Enhancement

Several studies have previously shown that it is possible to improve reconstruction accuracy through different approaches. For example, properly choosing a regularization method and an adequate parameter for the decoders can lead to better stimulus reconstruction accuracy of 10–20% (Crosse et al., 2016; Wong et al., 2018). Similarly, Montoya-Martínez et al. (2021) showed that by optimizing the number of electrodes used for reconstruction, it was possible to improve a median score of 0.17–0.22, which represents a gain of 29%. The improvement observed in our results in the challenging condition enables to increase the reconstruction accuracy from 0.09 to 0.11, which represents a gain of 22%. Therefore, the approach we present here yields an improvement in reconstruction accuracy comparable to other techniques reported in the literature.

### Acoustic Cues and Attention Switch Control

Spatial separation and voice differences are amongst the most important cues for auditory speech segregation. Teoh and Lalor (2019) improved auditory attention decoding accuracy by incorporating spatial attentional focus whereas Bednar and Lalor (2020) successfully reconstructed the spatial trajectory of a moving attended speech stream. The comparison of our results with the latter study is arduous due to methodological differences. Indeed, Bednar and Lalor’s (2020) approach to reconstructing the spatial trajectory of a constantly moving attended speaker differs from our method in two ways. First, they directly manipulated the spatial location of the sources and this information is contained in the stimuli themselves. In contrast, in our experiment, the speakers’ position was fixed and the attentional switches we captured with the behavioral responses were spurious rather than controlled. Second, Bednar and Lalor (2020) used a continuous variation of the location over time, whereas our behavioral account of attention is temporally restricted to three time windows corresponding to the three keyword positions. Between these keywords, we had to infer the participants’ focus of attention. Finally, while spatial location translates into continuous angles, our behavioral information is ternary (target, masker, or extraneous). Therefore, transposing the method introduced by Bednar and Lalor (2020) to our behavioral account of attention does not seem straightforward. Yet, such an approach would deserve further investigation, perhaps combining it with potential acoustic and linguistic correlates of attentional switches (such as fluctuations in local target-to-masker ratio, or overlap in semantic context across target and masker).

### Further Considerations and Conclusion

Results presented in the present study show that an enhancement of the stimuli reconstruction can be achieved in challenging situations where attention is modulated by voice cues such as F0 and VTL. By monitoring a participant’s attentional focus, it is possible to obtain a better reconstruction of the real attended speech and therefore a better cortical representation. The advantage of parametric voice manipulation, as introduced in this article, is that the listening difficulty can be controlled. By generating extremely challenging conditions, it is possible to approach listening situations that share similarities with those experienced by people with hearing loss. For instance, CI users do not seem to efficiently benefit from voice cues, such as F0 and VTL, to discriminate two speech streams (Gaudrain and Başkent, 2018; El Boghdady et al., 2019). This was also the case for the participants of this present experiment, under challenging listening conditions. However, to further understand how voice-based speech segregation is hindered in listeners with hearing loss, more studies need to be conducted either with actual HI or CI listeners (e.g., Somers et al., 2018; Paul et al., 2020), or using hearing loss or electrical stimulation simulations, which can allow researchers to focus on specific aspects of sensory degradation.

As mentioned earlier, our approach is based on a temporally restricted measure of attention. In fact, results showing that the shortest attentional scope (i.e., target windows) works better than the longest scope (i.e., segments) underline the need for a more precise temporal resolution. One way to extend this temporal measurement would be to ask participants to press a button whenever they listen to a target stimuli within the target stream similarly to Crosse et al. (2015) with a hit/false-alarm/miss scoring. This approach would provide a better temporal resolution even though it introduces a dual task. Furthermore, this approach would allow for a greater comprehension of attentional bottom-up cues in speaker reconstruction and decoding studies.

One of the major challenges in neural tracking studies is to identify, based on brain activity, the speaker that the participant is listening to in a cocktail party situation. Our results stress the importance of incorporating attentional-switch tracking in speech enhancement or noise-reduction algorithms in hearing-aids.

## Data Availability Statement

The data used in this study are available here: https://doi.org/10.5281/zenodo.5680384.

## Ethics Statement

Ethical review and approval was not required for the study on human participants in accordance with the local legislation and institutional requirements. The patients/participants provided their written informed consent to participate in this study.

## Author Contributions

M-PH, CM, and EG designed the experiment. M-PH and EP performed data worked on collecting data and logistics. M-PH and EG analyzed the data. M-PH, CM, EP, and EG wrote the manuscript. All authors contributed to the article and approved the submitted version.

## Conflict of Interest

CM was employed by the company Starkey France, S.a.r.l. The remaining authors declare that the research was conducted in the absence of any commercial or financial relationships that could be construed as a potential conflict of interest.

## Publisher’s Note

All claims expressed in this article are solely those of the authors and do not necessarily represent those of their affiliated organizations, or those of the publisher, the editors and the reviewers. Any product that may be evaluated in this article, or claim that may be made by its manufacturer, is not guaranteed or endorsed by the publisher.
